# Arsenic exposure in early pregnancy alters genome-wide DNA methylation in cord blood, particularly in boys

**DOI:** 10.1017/S2040174414000221

**Published:** 2014-04-14

**Authors:** K. Broberg, S. Ahmed, K. Engström, M. B. Hossain, S. Jurkovic Mlakar, M. Bottai, M. Grandér, R. Raqib, M. Vahter

**Affiliations:** 1 Institute of Environmental Medicine, Unit of Metals and Health, Karolinska Institutet, Stockholm, Sweden; 2 Department of Laboratory Medicine, Section of Occupational and Environmental Medicine, Lund University, Lund, Sweden; 3 International Centre for Diarrhoeal Disease Research Bangladesh (ICDDR,B), Dhaka, Bangladesh; 4 Department of Clinical Biochemistry, Faculty of Pharmacy, University of Ljubljana, Ljubljana, Slovenia; 5 Unit of Biostatistics, Institute of Environmental Medicine, Karolinska Institutet, Stockholm, Sweden

**Keywords:** 450 K, cancer, CpG, development, epigenetic, fetal

## Abstract

Early-life inorganic arsenic exposure influences not only child health and development but also health in later life. The adverse effects of arsenic may be mediated by epigenetic mechanisms, as there are indications that arsenic causes altered DNA methylation of cancer-related genes. The objective was to assess effects of arsenic on genome-wide DNA methylation in newborns. We studied 127 mothers and cord blood of their infants. Arsenic exposure in early and late pregnancy was assessed by concentrations of arsenic metabolites in maternal urine, measured by high performance liquid chromatography-inductively coupled plasma mass spectrometry. Genome-wide 5-methylcytosine methylation in mononuclear cells from cord blood was analyzed by Infinium HumanMethylation450K BeadChip. Urinary arsenic in early gestation was associated with cord blood DNA methylation (Kolmogorov–Smirnov test, *P*-value<10^–15^), with more pronounced effects in boys than in girls. In boys, 372 (74%) of the 500 top CpG sites showed lower methylation with increasing arsenic exposure (*r*_*S*_-values>−0.62), but in girls only 207 (41%) showed inverse correlation (*r*_*S*_-values>−0.54). Three CpG sites in boys (cg15255455, cg13659051 and cg17646418), but none in girls, were significantly correlated with arsenic after adjustment for multiple comparisons. The associations between arsenic and DNA methylation were robust in multivariable-adjusted linear regression models. Much weaker associations were observed with arsenic exposure in late compared with early gestation. Pathway analysis showed overrepresentation of affected cancer-related genes in boys, but not in girls. In conclusion, early prenatal arsenic exposure appears to decrease DNA methylation in boys. Associations between early exposure and DNA methylation might reflect interference with *de novo* DNA methylation.

## Introduction

The environment of the developing child is an important determinant of disease susceptibility in later life.^1^ This phenomenon may be mediated through epigenetic changes, such as alterations of DNA methylation, which alter developmental programming.^2^ ^–^ ^5^ Lately, it has become increasingly clear that toxic chemicals can also affect DNA methylation.^6^ ^–^ ^8^

Arsenic, which frequently occurs at elevated concentrations in drinking water worldwide, is a potent carcinogen, a general toxicant and an endocrine disrupter.^9^ ^,^ ^10^ Besides the accumulating evidence for associations between *in utero* arsenic exposure and impaired fetal and child health and development,^11^ ^–^ ^14^ both human and experimental animal studies indicate higher risk for cancer, cardiovascular effects and increased mortality later in childhood or adulthood when exposure starts prenatally or shortly after birth.^15^ ^–^ ^20^ These results suggest persistent long-term effects of early-life changes, possibly through alterations of DNA methylation at the 5-methylcytosine position. Studies on epigenetic effects of arsenic mainly have concerned changes in DNA methylation in relation to cancer development, which is closely related to DNA methylation instability.^21^ ^–^ ^25^ In particular, arsenic-related changes in gene methylation status have been proposed to silence tumor-suppressor genes, possibly eading to long-term changes in the activity of genes controlling cell transformation.^21^ These results suggest that arsenic has epigenetic effects related to its carcinogenicity. Genome-wide DNA methylation has been assessed in a small group (*n*=16) of adults.^26^ Studies on the effects of arsenic exposure on DNA methylation in newborns are few and have generally examined non-gene-related markers of DNA methylation.^27^ ^–^ ^29^ A recent study by Koestler *et al*.^30^ investigated the association between low maternal arsenic exposure measured in late pregnancy and genome-wide effects on 5-methylcytosine methylation in cord blood. Therefore, the aim of the present study was to evaluate cord blood DNA methylation of gene-related CpG sites, covering a large part of the whole epigenome, in relation to a large range of maternal arsenic exposure in both early and late pregnancy. Considering the increasing evidence for sexual dimorphism both for programming trajectories and in response to the environmental insults,^31^ including arsenic,^29^ ^,^ ^32^ we evaluated the results in female and male newborns separately.

## Methods

### Study area and design

The study area, Matlab, is a rural area southeast of Dhaka, where the International Centre for Diarrhoeal Disease Research, Bangladesh (ICDDR,B), runs a health and demographic surveillance system. In a population-based screening in 2002–2003, we found a total range of <1 to 3640 µg/l, with 70% of the tube wells in Matlab exceeding the WHO-recommended limit of arsenic in drinking water of 10 µg/l.^33^ In an effort to decrease adverse health effect because of polluted drinking water, most families (>95%) have had a well drilled in their yard past few decades ago, or obtain water from community-owned wells.^34^ Unfortunately, a considerable fraction of those wells were later found to contain arsenic, because of the hydrogeological conditions, and this has been related to impaired fetal growth and increased infant morbidity and mortality. Other toxic exposures are low. This is a very pristine rural area of Bangladesh, with very little environmental pollution, for example, essentially no cars.

The study was nested in a large randomized population-based food and micronutrient supplementation trial (MINIMat) in pregnancy.^35^ Pregnancy was identified by urine test, usually in gestational week (GW) 8. Women who tested positive were advised to visit the health facility for confirmation of pregnancy by ultrasound. The current study is based on 127 women who delivered at any of the health centers before early afternoon (5:00 am–2:30 pm, owing to logistics in transferring samples to the laboratory) from May 2003 to June 2004. According to the questionnaire, all women were non-smokers, while 73 (58%) of the women chewed betel leaves (6 out of 73 women reported betel chewing with tobacco) during pregnancy.

Birth weight was measured mostly within 24 h of delivery (all within 72 h), using electronic scales. Socioeconomic status was based on family assets using data from the health demographic surveillance system database.^36^ Participants gave written informed consent and the study was approved by the Research Review Committee and Ethical Review Committee of ICDDR,B and the Regional Ethics Committee at the Karolinska Institutet, Sweden.

### Arsenic exposure

Arsenic exposure was based on the sum concentration of arsenic metabolites [inorganic arsenic, methylarsonic acid (MMA) and dimethylarsinic acid] in spot urine samples collected, as previously described.^37^ This is an established biomarker that reflects exposure to inorganic arsenic from all sources. The half-life of arsenic in the body is 3–4 days, which is why spot urine reflects the ongoing exposure. However, because the exposure in the area is mainly from drinking water, with some contribution from rice, the main staple food, the exposure is fairly stable over time.^38^ Because of the local custom that young women, especially first-time mothers, move to their parents’ home for delivery, we evaluated arsenic exposure in both early and late pregnancy, using urine samples from 127 women that were collected between GW 5 and 14 (median GW 8) and between GW 26 and 36 (median GW 30), respectively. The relative amounts of the different metabolites in urine were used as markers of methylation efficiency.^39^ The reason is that we recently found that the efficiency of arsenic methylation increases very early in pregnancy.^38^ In particular, the percentage of MMA, which has been associated with increased risk of toxic effects in adults,^40^ ^,^ ^41^ decreased very early in pregnancy. The arsenic metabolite concentrations were measured using hydride generation atomic absorption spectrophotometry with a detection limit of 1.3±0.27 µg/l. All arsenic concentrations were adjusted for variation in urine dilution by specific gravity to the average value, 1.012 g/ml.

### DNA isolation and epigenetic analysis

Cord blood mononuclear cells were separated by Ficoll (Pharmacia-Upjohn) density gradient centrifugation. The epigenetic analysis has been described in detail elsewhere.^42^ In brief, 1 µg DNA (50 ng/µl) was bisulfite-treated (Zymo Research). The DNA samples were randomized for gender and arsenic exposure on two 96-well plates for analysis with the Infinium HumanMethylation450K BeadChip (lllumina). All chips were from the same batch. We also included four controls in duplicate, with the duplicate positioned on different 96-well plates and different BeadChips. Three of the control samples were DNA extracted from blood and one was demethylated DNA (Zymo Research). The SCIBLU facility (Lund, Sweden) used 200 ng bisulphite-treated DNA for hybridization to the BeadChip. Unsupervised hierarchial cluster analysis showed that all duplicates of the control samples clustered together.

For all samples, the number of detected CpGs (*P*-value=0.01) were at least >98%. All 1500 probes (the top 500 selected for all children, top 500 selected for boys and top 500 selected for girls) performed very well with a detection *P*-value of at least 0.01 (the absolute majority *P*<0.0001) among 80% of the samples. In an ongoing study on DNA samples from whole blood we are evaluating the concordance between results from the HumanMethylation450K BeadChip and pyrosequencing. We found a correlation of 0.85–0.92 (*P*>0.0001) for four different CpG sites tested for both methods.

Measurement of LINE1 methylation, as a marker for global methylation status, was performed on bisulphite-treated cord blood DNA from a subgroup of 80 newborns (median of maternal arsenic in urine at GW 8 was 66 µg/l, range 3–740 µg/l): 40 boys and 40 girls. Measurement of four CpG sites in the LINE1 sequence was analyzed by the use of pyrosequencing, as described previously.^43^

### Statistical analysis

Details of the statistical analyses were reported previously.^7^ Missing beta values (0.1%) were imputed using *k*-nearest neighbor imputation (*k*=10). Principal component analysis (PCA), using the R package swamp, was used to capture the major directions of variation in the data and influencing factors. For each of the top principal components, we fitted a univariate linear model with each of the sample annotations as regressors. The log10 *P*-values from the models’ *F*-statistics were plotted as a heat map. The results showed that the analysis plate (two 96-well plates) was associated with methylation levels in the first (*P*=10^−6^) and third components (*P*<10^−10^). We removed the plate influence at each CpG site by using the residuals from the initial linear regression model of methylation with the analysis plate as regressor. The residuals of the linear model added to the total mean before correction became the new data for each CpG site. In this way, the methylation levels of the sites were not influenced by the analysis plate.

We first evaluated whether the maternal arsenic exposure (urinary arsenic) overall was associated with genome-wide DNA methylation by performing 482,421 separate linear regression models, one for each CpG site, with methylation as the dependent variable and arsenic concentration as the independent variable. The range of arsenic was rescaled to vary between 0 and 1 to minimize the potential effects of skewed data. We tested whether the slope was statistically significant in all 482,421 models. Had there been no effect of arsenic on any CpG site, then the slopes would result from mere sampling error. In this case, the *P*-values would be distributed uniformly over the 0–1 range.

The associations of CpG site methylation fractions with urinary arsenic were then determined using Spearman correlation. Resulting *P*-values were corrected for multiple testing by the Benjamini–Hochberg method to obtain false discovery rates.

Some of the associations between DNA methylation and maternal urinary arsenic were non-linear, but after log2 transformation of the arsenic concentrations the associations showed linear patterns, and linear regression analyses were applied. We did not observe any typical genotype-related clustering of DNA methylation when visually inspecting the associations between arsenic and DNA methylation. In multivariable-adjusted analysis, we controlled for the maternal characteristics age, body mass index (BMI) (at GW 8), SES, as well as GW at urine collection, gestational age at delivery and fetal sex (except for analyses stratified for sex). In sensitivity analysis, we additionally adjusted for birth weight, betel chewing including with tobacco and cadmium.

For the pathway analysis, we used the Ingenuity Pathway Analysis Tool (Ingenuity H Systems). We selected the top 500 gene-annotated CpG sites associated with arsenic in maternal urine in boys and girls separately (from Illumina). Differentially methylated genes are mapped to genetic networks/diseases available in the Ingenuity Pathway Knowledge Base and then ranked by score.

## Results

### Background data

The studied women were on average 25.3 years old and with a BMI of 20.4 kg/m^2^ (Table 1). The gestational age at birth was 39 weeks on average: 12 births (six boys and six girls) being premature (range 34.4–36.7 weeks at birth). Boys had slightly higher birth weight (2838 g) than girls (2724 g). The median maternal urinary arsenic concentrations were 66 μg/l in GW 8 and 89 μg/l in GW 30, with wide variations. Maternal urinary arsenic concentration did not differ by sex of the child. Women in the study sample were to a larger extent nulliparous, had higher SES and had children with somewhat higher birth weight, compared with the total cohort of 1729 pregnant women studied for arsenic exposure.

**Table 1:** Characteristics of the 127 mother–child pairs and all other women enrolled in the MINIMat trial^a^

	Study sample (*n*=127)	All women (*n*=1729)
Variables	Mean±s.d. or median (5–95 percentiles) or *n* (%)	Mean±s.d. or median (5–95 percentiles) or *n* (%)
Maternal characteristics		
Maternal age (years)	25.3±5.9	26.4±5.9
BMI (kg/m^2^) at GW 8	20.4±3.0	20.1±2.7
Parity (no. of children)	1.1±1.4	1.4±1.4
0/⩾1	54 (43%)/73 (57%)	548 (32%)/1179 (68%)
Gestational age (week, GW)	38.7±1.8	38.8±2.1
<37/⩾37	12 (10%)/112 (90%)	254 (15%)/1444 (85%)
Betel chewing during pregnancy		
Yes/No	73 (58%)/52 (42%)	1150 (68%)/531 (32%)
Socioeconomic status		
Lowest	19 (15%)	315 (18%)
Lower middle	12 (9%)	349 (20%)
Middle	24 (19%)	353 (20%)
Upper middle	36 (28%)	350 (20%)
Highest	36 (28%)	362 (21%)
Urinary arsenic GW 8 (µg/l)^b^	66 (20–457)	66 (17–466)^c^
Inorganic arsenic (%)^d^	13.8±5.5	na
MMA (%)^d^	10.06±3.6	na
DMA (%)^d^	76.12±7.5	na
Urinary arsenic GW 30 (µg/l)^d^	89 (18–562)	80 (19–610)^c^
Infant characteristics		
Birth weight (g)	2780±395	2709±409
Low birth weight (<2500 g)	26 (21%)	504 (25%)
Sex (% girls)	65 (51%)	845 (49%)

BMI, body mass index; GW, gestational week; MMA, methylarsonic acid; DMA, dimethylarsinic acid.

a Enrollment in the MINIMat trial from October 2002 to October 2003.

b Adjusted to average specific gravity of 1.012 g/ml.

c In MINIMat, urinary arsenic was measured at GW 8 (*n*=1729) and GW 30 (*n*=1031).

d Percent of total metabolite concentration in urine. Urinary arsenic refers to inorganic arsenic and its methylated metabolites in urine.

### Maternal arsenic exposure and genome-wide DNA methylation

The PCA showed that maternal arsenic exposure in early gestation (GW 8) was associated with cord blood DNA methylation in the eighth component and, to a lesser extent, in the fourth component (Supplementary Figure S1). Arsenic exposure measured in late gestation (GW 30) showed weaker association with DNA methylation than that in GW 8.

**Fig. 1:**
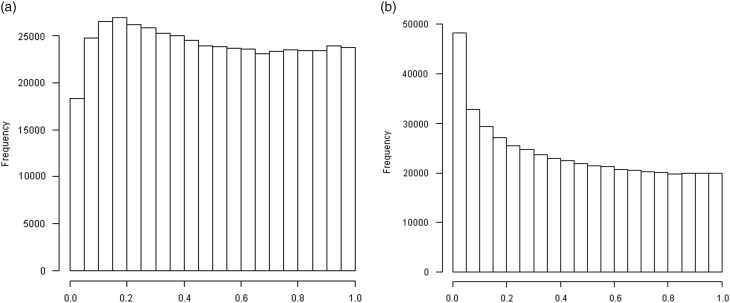
Distribution of the *P*-values for the coefficient associated with maternal urinary arsenic concentrations in early gestation from linear regression analysis of CpG methylation (*n*=482,421 sites) in cord blood. (*a*) Cord blood from girls, (*b*) Cord blood from boys.

We first evaluated whether maternal arsenic exposure in early gestation was associated with newborn genome-wide DNA methylation by analyzing all CpG sites in cord blood *v*. arsenic concentrations in maternal urine in GW 8 (482,421 separate models). Low *P*-values were more frequent than expected from a uniform distribution (Kolmogorov–Smirnov test, *P*-value<10^–15^, Supplementary Figure S2), and there were marked differences between sexes; significant effects of arsenic were considerably more pronounced in boys compared with girls (Fig. 1). The proportion of small *P*-values was greater than expected in boys (*P*-value<0.0001), but not in girls (*P*-value=0.99).

**Figure 2:**
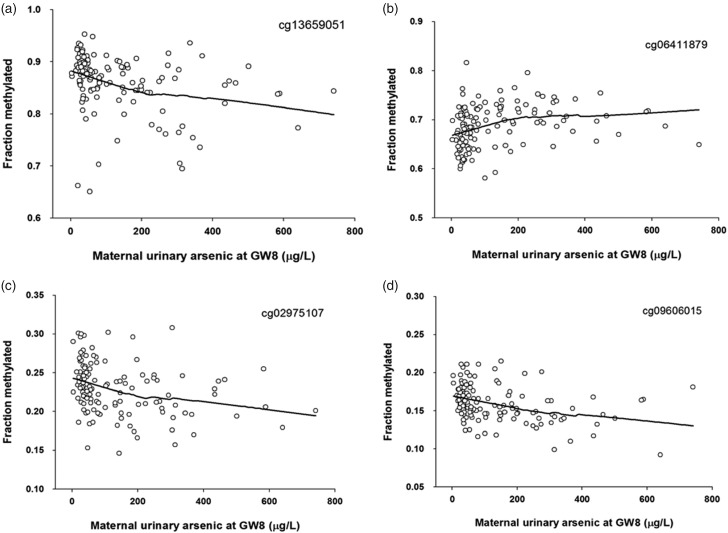
Scatterplot of the fractions (0 to 1) of methylation of the CpG sites in cord blood and the concentrations of arsenic metabolites in maternal urine in early pregnancy (gestational week 8) for: (*a*) cg13659051 and (*c*) cg02975107, among top five CpG sites in boys; and (*b*) cg06411879 and (*d*) cg09606015, among top five CpG sites in girls. The associations are indicated by Lowess lines.

We selected the top 500 CpG loci with the lowest *P*-values based on their association with urinary arsenic levels in GW 8 and found hypomethylation with increasing arsenic exposure for 277 (55%) sites, and hypermethylation for 223 (45%) sites (all infants, test if more hypomethylation than expected gave *P*=0.018). There were major sex differences: among boys 372 (74%) of the top 500 sites showed hypomethylation with increasing arsenic exposure, compared with only 207 (41%) among girls (difference between boys and girls *P*-value<0.001, Fisher’s exact test). We found no single overlap between the top 500 arsenic-related sites in boys and those in girls. The 20 CpG sites with the strongest association with urinary arsenic in GW 8 in boys and girls are listed in Table 2. Generally, stronger associations were found for boys compared with girls, as estimated from correlation coefficients and *P*-values; three CpG sites in boys (cg15255455, cg13659051 and cg17646418), but none in girls, were significantly associated with arsenic exposure after adjustment for multiple comparisons (Table 2). There were stronger associations between DNA methylation and maternal arsenic exposure measured in early than in late gestation, but the directions were the same. Examples of the associations are given in Fig. 2, which indicate that the effects of arsenic on DNA methylation start at low exposure levels, well below 100 µg/l.

**Table 2:** Top 20 CpG sites with the strongest correlations (*r*_*S*_) with maternal urinary arsenic concentrations in gestational week (GW) 8 by child sex, as well as regression analysis (GW 8)^a^

CpG sites	Chr	Gene	*r*_*S*_ (GW 8)	*P*-value	FDR^b^	*r*_*s*_ (GW 30)	*P*-value	FDR^b^	Regression β GW 8 (95%CI)^c^	*P*-value
Boys										
cg15255455	19	*PLIN5*	−0.62	1.3×10^–7^	0.031	−0.41	<0.001	0.99	−0.016 (−0.022, −0.0092)	<0.001
cg13659051	9	*LRRC26*	−0.62	1.3×10^–7^	0.031	−0.25	0.053	0.99	−0.018 (−0.026, −0.0098)	<0.001
cg17646418	6	*RPS6KA2*	−0.61	2.3×10^–7^	0.036	−0.22	0.093	0.99	−0.011 (−0.017, −0.0066)	<0.001
cg02975107	17		−0.58	1.1×10^–6^	0.080	−0.42	<0.001	0.99	−0.0099 (−0.014¸ −0.0050)	<0.001
cg00592460	6		−0.57	1.3×10^–6^	0.080	−0.29	0.022	0.99	−0.020 (−0.027¸ −0.012)	<0.001
cg05114100	X	*LUZP4*	−0.57	1.4×10^–6^	0.080	−0.34	0.0073	0.99	−0.012 (−0.018, −0.0059)	<0.001
cg23727087	12		−0.57	1.7×10^–6^	0.080	−0.15	0.24	0.99	−0.0089 (−0.013, −0.0046)	<0.001
cg17158414	2	*KRTCAP3*	−0.57	1.7×10^–6^	0.080	−0.39	0.0018	0.99	−0.027 (−0.041, −0.013)	<0.001
cg24042517	17	*HOXB9*	−0.57	1.7×10^–6^	0.080	−0.33	0.0084	0.99	−0.010 (−0.01, −0.0055)	<0.001
cg12124733	X	*DDX26B*	0.57	1.8×10^–6^	0.080	0.30	0.018	0.99	0.0069 (0.00086, 0.013)	0.026
cg19802390	1		−0.56	1.9×10^–6^	0.080	−0.18	0.16	0.99	−0.023 (−0.034, −0.011)	<0.001
cg24456094	6	*MDGA1*	−0.56	2.1×10^–6^	0.080	−0.30	0.015	0.99	−0.012 (−0.018, −0.0054)	0.001
cg15479387	6	*HLA-F-AS1*	−0.56	2.2×10^–6^	0.080	−0.27	0.032	0.99	−0.0047 (−0.0069, −0.0025)	<0.001
cg25938735	8		−0.56	2.5×10^–6^	0.085	−0.22	0.079	0.99	−0.014 (−0.019, −0.0078)	<0.001
cg20415517	11	*BRSK2*	−0.55	2.8×10^–6^	0.085	−0.28	0.025	0.99	−0.013 (−0.018, −0.0072)	<0.001
cg12094808	15	*FRMD5*	−0.55	2.8×10^–6^	0.085	−0.30	0.016	0.99	−0.011 (−0.016, −0.0059)	<0.001
cg14920289	14	*SLC22A17*	−0.55	3.6×10^–6^	0.095	−0.36	0.0039	0.99	−0.0098 (−0.014, −0.0052)	<0.001
cg21097283	5	*OTP*	0.55	3.9×10^–6^	0.095	0.25	0.052	0.99	0.0093 (0.0045, 0.014)	<0.001
cg17322444	19	*GPATCH1*	0.54	4.5×10^–6^	0.095	0.25	0.045	0.99	0.0044 (0.0026, 0.0061)	<0.001
cg24680320	19		−0.54	4.5×10^–6^	0.095	−0.29	0.019	0.99	−0.0030 (−0.0043, −0.0017)	<0.001
Girls										
cg22614624	2	*DTYMK*	0.55	3.2×10^–6^	0.52	0.24	0.053	0.99	0.012 (0.0053, 0.019)	0.001
cg08421080	12	*RIMBP2*	0.54	4.5×10^–6^	0.52	0.39	0.0014	0.99	0.0085 (0.0010, 0.015)	0.026
cg02240066	2		−0.54	5.0×10^–6^	0.52	−0.05	0.67	0.99	−0.0052 (−0.0079, −0.0025)	<0.001
cg09606015	3	*ATP11B*	−0.54	5.3×10^–6^	0.52	−0.16	0.22	0.99	−0.0072 (−0.011, −0.0032)	0.001
cg06411879	10	*NEBL*	0.54	5.4×10^–6^	0.52	0.27	0.029	0.99	0.013 (0.0040, 0.021)	0.005
cg23385248	15		−0.53	9.8×10^–6^	0.61	−0.31	0.012	0.99	−0.013 (−0.020, −0.0063)	<0.001
cg08962682	12	*RIMKLB*	−0.52	1.5×10^–5^	0.61	−0.22	0.074	0.99	−0.0036 (−0.0061, −0.0011)	0.005
cg05173913	17	*C17orf50*	−0.50	3.0×10^–5^	0.61	−0.25	0.043	0.99	−0.0062 (−0.010, −0.0017)	0.007
cg21587006	10		0.50	3.0×10^–5^	0.61	0.34	0.0053	0.99	0.054 (0.0024, 0.10)	0.040
cg06584028	2	*GFPT1*	−0.50	3.4×10^–5^	0.61	−0.20	0.12	0.99	−0.0053 (−0.0080, −0.0027)	<0.001
cg04361852	11	*UCP2*	−0.49	3.5×10^–5^	0.60	−0.14	0.27	0.99	−0.0043 (−0.0072, −0.0013)	0.005
cg00384577	19	*ITPKC*	−0.48	4.9×10^–5^	0.60	−0.19	0.13	0.99	−0.0052 (−0.0078, −0.0024)	<0.001
cg14862981	5		−0.48	5.3×10^–5^	0.60	−0.14	0.27	0.99	−0.0027 (−0.0048, −0.00064)	0.01
cg09005548	6	*ZBTB22*	0.48	5.6×10^–5^	0.60	0.23	0.061	0.99	0.011 (0.0053, 0.016)	<0.001
cg14673387	10		−0.48	5.8×10^–5^	0.60	−0.098	0.44	0.99	−0.0033 (−0.0049, −0.0016)	<0.001
cg22328208	8	*TSPYL5*	−0.48	6.2×10^–5^	0.60	−0.29	0.017	0.99	−0.015 (−0.025, −0.0043)	0.007
cg07276007	11	*AP2A2*	0.48	6.8×10^–5^	0.60	0.38	0.0017	0.99	0.0092 (0.0012, 0.017)	0.024
cg07936037	6	*SSR1*	−0.48	6.9×10^–5^	0.60	−0.15	0.24	0.99	−0.0026 (−0.0043, −0.00082)	0.005
cg26726230	17	*SLC38A10*	0.47	7.0×10^–5^	0.60	0.038	0.76	0.99	0.024 (0.011, 0.036)	<0.001
cg01094684	19	*UQCR*	−0.47	7.1×10^–5^	0.60	−0.28	0.021	0.99	−0.0033 (−0.0053, −0.0012)	0.002

Chr, chromosome number; FDR, false discovery rate; CI, confidence intervals In the regression analysis, the arsenic exposure variable was log2-transformed.

a Correlations in GW 30 are also shown.

b *P*-value adjusted for FDR.

c Adjusted for mother age, body mass index, gestational age at birth, socioeconomic status, exact gestational weeks at urine collection.

For the CpG sites in Table 2, only 1 (cg22614624) of the 40 sites contained an single-nucleotide polymorphism (SNP) within ⩽10 bp from the query site (Supplementary Table S2). This SNP has a minor allele frequency of 0.349, based on very little data and none from Asian populations (www.ncbi.nlm.nih.gov/projects/SNP). We also analyzed the data without the sex chromosomes, but this did not influence the results. The reported *P*-values were still significant as well as the order of the top SNPs reported.

In the subsequent linear regression analyses (log2-transformed urinary arsenic concentrations in GW 8), the associations between CpG methylation and arsenic exposure in early gestation were very robust when taking other potentially influential covariates into account (Table 2). Apart from GW of urine collection, few covariates were even close to significant in the models and their inclusion did not markedly change the estimates for arsenic. In a sensitivity analysis, we additionally adjusted for birth weight, betel chewing and maternal cadmium in blood, but this only marginally changed the effect estimates. Among the 20 top sites in boys, the effect of arsenic on the degree of CpG methylation ranged between 0.3 and 2.7% for a doubling of arsenic concentrations in maternal urine (µg/l) in early pregnancy. Arsenic caused in boys mainly lower methylation (all 12 sites with >1% change involved hypomethylation). The corresponding range for girls was 0.3–5.4% (of the seven sites with >1% change, two showed lower and five higher methylation).

We performed analysis of LINE1 in a subgroup of 40 boys and 40 girls. There were no significant differences in LINE1 methylation (expressed as mean percentage of methylation of the four CpG sites) between the sexes (mean 68.0% in boys and 68.3% in girls, *P*=0.75, ANOVA). Furthermore, there were no significant associations between urinary arsenic in GW 8 and LINE1 methylation in all newborns (*P*=0.86, adjusted for the same covariates as in the analysis above for individual CpG sites *P*=0.59, ANCOVA), or in boys or girls separately (boys: *P*=0.73, adjusted *P*=0.59; girls: *P*=0.46, adjusted *P*=0.45).

We additionally stratified the regression analyses by the percentage of MMA in urine (GW 8), as a measure of maternal arsenic methylation efficiency, mothers with relatively high percentage of MMA were considered to have poorer methylation efficiency (Supplementary Table S3). For several of the top 20 sites, the arsenic-related effect on CpG methylation was markedly stronger in newborns to mothers with poor arsenic methylation.

#### Arsenic exposure in early gestation and epigenetic changes related to disease pathways

We performed pathway analysis of the top 500 gene-annotated CpG sites for which methylation was affected by arsenic in boys and girls. In both boys and girls, there was overrepresentation of networks related to embryonic, organic and cellular development (Table 3). In contrast, cancer-related networks were found to be overrepresented in boys (146 genes, Supplementary Table S4), but not in girls. Of the 146 genes, 67% were hypomethylated with increasing arsenic exposure, the remaining were hypermethylated. When searching for related diseases that were overrepresented in boys, cancer was the first one listed (*P*-value=4.4×10^–5^) followed by reproductive system diseases, hematological diseases, hereditary disorder, and skeletal and muscular disorders. In girls, inflammatory disease ranked the highest (*P*-value=2.7×10^–5^), followed by neurological diseases, skeletal and muscular disorders, endocrine system disorders and gastrointestinal diseases.

**Table 3:** Pathway analysis^a^, stratified for sex, between maternal urinary arsenic concentrations in early gestation CpG methylation in cord blood

Boys
Top networks
Embryonic development, organ development, organ morphology
Cancer, drug metabolism, molecular transport
Antimicrobial response, cell-to-cell signaling, embryonic development
Neurological disease, hematological disease, hereditary disorder
Cellular development growth and proliferation, reproductive system development and function
Cardiovascular disease, cancer, hematological disease
Embryonic development, organismal development, cell signaling
Cancer, cellular development, cardiovascular disease
Organ morphology, cancer, reproductive system disease
Cell cycle, hereditary disorder, ophthalmic disease
Top diseases and disorders (number of genes, *P*-value)
Cancer (146, 4.4×10^–5^)
Reproductive system disease (46, 4.4×10^–5^)
Hematological disease (43, 1.2×10^–4^)
Hereditary disorder (20, 1.2×10^–4^)
Skeletal and muscular disorders (14, 4.8×10^–4^)
Girls
Top networks
Embryonic development, nervous system development and function, organ development
Infectious disease, cellular development, cellular growth and proliferation
Gene expression, cell cycle, DNA replication, recombination and repair
Connective tissue disorders, tissue morphology, connective tissue development and function
Cell cycle, connective tissue disorders, infectious disease
Respiratory system development, cell-to-cell signaling, hematological development and function
Post-translational modification, cell-to-cell signaling, cellular movement
Cellular development, embryonic development, nervous system development and function
Developmental disorder, hereditary disorder, metabolic disease
Hereditary disorder, neurological disease, connective tissue disorders
Top diseases and disorders (number of genes, *P*-value)
Inflammatory disease (20, 2.7×10^–5^)
Neurological disease (55, 2.7×10^–5^)
Skeletal and muscular disorders (66, 2.7×10^–5^)
Endocrine system disorders (42, 2.0×10^–4^)
Gastrointestinal disease (42, 2.0×10^–4^)

a The input data to the Ingenuity Pathway Analysis software was the top annotated genes from the top 500 scoring CpG sites in cord blood correlated to arsenic in maternal blood in boys and girls, respectively.

We performed linear regression analysis of the top 10 CpG sites identified among the 146 genes associated with cancer in boys (and in girls for comparison). The results (Table 4) show that the associations in boys were robust in the models, and that methylation in these sites was not associated with arsenic in girls. Three CpG sites (cg13659051, cg24042517 and cg20415517) associated with cancer-related genes were also among the top sites in Table 2: *LRRC26*, *HOXB9* and *BRSK2*.

**Table 4:** Top 10 cancer-related CpG sites in boys (and girls for comparison) with the strongest correlations (*r*_*S*_) with maternal arsenic urinary concentrations in gestational week 8

CpG sites	Chr	Gene	*r*_*S*_	*P*-value	FDR^a^	β (95%CI)^b^	*P*-value
Boys							
cg13659051	9	*LRRC26*	−0.62	1.3×10^–7^	0.031	−0.018 (−0.026, −0.0098)	<0.001
cg24042517	17	*HOXB9*	−0.57	1.7×10^–6^	0.080	−0.010 (−0.015, −0.0055)	<0.001
cg20415517	11	*BRSK2*	−0.56	2.8×10^–6^	0.085	−0.013 (−0.018, −0.0072)	<0.001
cg04517524	14	*ASB2*	−0.55	5.2×10^–6^	0.095	−0.022 (−0.032¸ −0.011)	<0.001
cg26243679	6	*FRK*	−0.52	1.2×10^–5^	0.10	−0.014 (−0.021, −0.0069)	<0.001
cg08660959	6	*TNXB*	−0.52	1.2×10^–5^	0.10	−0.0046 (−0.0069, −0.0021)	<0.001
cg06611850	3	*DPPA2*	−0.52	1.3×10^–5^	0.10	−0.014 (−0.021, −0.0066)	<0.001
cg21050076	19	*GCDH*	−0.52	1.3×10^–5^	0.10	−0.0092 (−0.013, −0.0048)	<0.001
cg21856067	20	*MACROD2*	−0.52	1.3×10^–5^	0.10	−0.0053 (−0.0081, −0.0025)	<0.001
cg24698211	5	*FGF18*	−0.52	1.5×10^–5^	0.10	−0.016 (−0.024, −0.0069)	<0.001
Girls							
cg13659051	9	*LRRC26*	−0.20	0.10	0.61	−0.00092 (−0.012, 0.011)	0.88
cg24042517	17	*HOXB9*	−0.14	0.26	0.68	−0.0019 (−0.0086, 0.0049)	0.58
cg20415517	11	*BRSK2*	0.10	0.41	0.75	0.0037 (−0.0051, 0.012)	0.41
cg04517524	14	*ASB2*	0.17	0.16	0.63	−0.00038 (−0.017, 0.016)	0.96
cg26243679	6	*FRK*	0.0065	0.95	0.98	0.0038 (−0.0043, 0.011)	0.36
cg08660959	6	*TNXB*	−0.07	0.54	0.83	0.00013 (−0.0035, 0.0038)	0.94
cg06611850	3	*DPPA2*	−0.15	0.25	0.67	0.00028 (−0.0089, 0.0094)	0.95
cg21050076	19	*GCDH*	−0.06	0.63	0.86	−0.0029 (−0.0085, 0.0028)	0.32
cg21856067	20	*MACROD2*	−0.22	0.85	0.95	−0.0018 (−0.0066, 0.0029)	0.44
cg24698211	5	*FGF18*	−0.18	0.13	0.62	−0.0018 (−0.012, 0.0089)	0.74

Chr, chromosome number; FDR, false discovery rate; CI, confidence intervals. In the regression analysis arsenic exposure variable was log2-transformed.

a *P*-value adjusted for FDR.

b Adjusted for mother’s age, body mass index, gestational age at birth, socioeconomic status, exact gestational weeks at urine collection.

## Discussion

This first study of sex-differential genome-wide cord blood DNA methylation in relation to arsenic exposure provides strong evidence that maternal arsenic exposure in early gestation is associated with modified DNA methylation in the newborn child. For many of the CpG sites, arsenic exposure changed the DNA methylation by several percent, and the changes started at low exposure levels, below 50 µg/l in the urine. At the genome-wide DNA methylation level, the associations with arsenic exposure were much more evident in boys than in girls. In particular, arsenic seemed to induce hypomethylation in boys; that is, in 74 of the top 500 CpG sites methylation decreased with increasing arsenic exposure. In a subgroup of the newborns, we evaluated whether the decrease in methylation with increasing arsenic exposure in boys, reflects a general genomic feature, such as interference of arsenic with the methyl donor pool. However, we did not find any overall effect of arsenic on global methylation in either of the sexes. Furthermore, we did not observe multiple probes affected for individual genes but rather strong effects on individual CpG sites in relation to arsenic. This observation, together with the data for global methylation, suggests that arsenic affects accessible areas of the chromatin with, certain structural features, or impacts on specific transcription factors or other chromatin-associated proteins. The pathway analysis showed enrichment of DNA methylation changes in cancer-related genes in boys, but not in girls. This is interesting considering the reported sex differences in cancer development in mice following prenatal arsenic exposure, with later-life occurrence of liver carcinoma and adrenal cortical adenoma in male, but not female, offspring.^44^ A further observation is noteworthy in relation to our data that the follow-up of the male liver DNA showed significant reduction in methylation of GC-rich regions.^19^ In contrast, mice that were continuously exposed also after birth showed much less sex differences in cancer development,^45^ which is in agreement with the observed marked increase in liver cancer mortality in both boys and girls exposed to arsenic through drinking water in northern Chile as young children, but not in those exposed before birth.^15^ It should be noted that we have identified arsenic-related impaired fetal growth in boys,^46^ although postnatal growth seems to be more affected in girls.^14^ Here we identified different processes involved in development, as the top pathways for arsenic-related DNA methylation change in both boys and girls. One can speculate that arsenic might interfere with already existing sex-specific gene expression early in life^31^ as seen in mice exposed to arsenic,^47^ or alternatively it causes sex-specific DNA methylation for genes that should not differ between the sexes. Thus, it is essential to follow-up on the epigenetic effects of arsenic as the children grow.

The much stronger associations with arsenic exposure in early compared with late gestation may reflect interference with the *de novo* DNA methylation patterns established in early fetal development.^5^ The rapid upregulation of the second step in arsenic methylation, resulting in a decrease in the percentage of the most toxic metabolite MMA very early in pregnancy,^38^ may further contribute to the observed weaker epigenetic associations with late gestation exposure levels. Indeed, our data also suggest that a low capacity for methylation of arsenic, resulting in a higher fraction of urinary MMA, a known susceptibility factor for arsenic toxicity,^40^ ^,^ ^41^ was associated with stronger epigenetic effects. However, the results in relation to methylation capacity need to be followed up, as they were based on a small number of children in each comparison group. Koestler *et al*.^30^ also found the effects of prenatal exposure to arsenic on cord blood methylation; however, in contrast to our data, they found increasing methylation with increasing arsenic exposure for most top sites. The results are difficult to compare with ours as the women in their study had markedly lower exposure (median maternal urinary arsenic was 4 *v*. 66 µg/l in our study) measured late in pregnancy, and moreover they did not stratify their results for sex.

The top sites associated with arsenic might represent possible new candidates for arsenic epigenome toxicity. In boys, three CpG sites associated with different genes (*PLIN5*, *LRRC26* and *RPS6KA*), not previously linked to arsenic, were significantly hypomethylated in relation to arsenic exposure. Perilipin 5 (PLIN5) is a lipid metabolism-related protein; overexpression of *PLIN5* in rat skeletal muscle promoted oxidative gene expression and intramyocellular lipid content.^48^ Heart-specific overexpression of *PLIN5* in mice was associated with increased triglyceride concentrations and strong increased expression of oxidative-induced genes via NF-E2-related factor 2 antioxidative pathway.^49^ *LRRC26* is a protein associated with calcium-activated potassium channels, so-called BK channels. It is normally expressed not only in the fetal brain and thymus, but also in prostate cancer where its expression actually reduced tumor growth and metastasis.^50^ *RPS6KA* is a type of serine/threonine kinase, implicated in controlling cell growth and differentiation and it has been identified as a risk marker for colon and rectal cancer.^51^ In addition, *HOXB9* and *BRKS2* are worth mentioning as they were among the cancer-related genes and among the top 20 genes for boys. *HOXB9*, a class I homebox gene, is overexpressed in breast cancer and it induces an epithelial-to-mesenchymal transition, a key factor in metastasis.^52^ *BRSK2* is an AMP-activated protein kinase and a multifunctional regulator of cell-cycle progression. Further studies are needed to examine the DNA methylation of these newly identified genes related to arsenic exposure later in life and in relation to cancer risk. Moreover, as we measure changes in DNA methylation in blood, they need to be followed up in relation to potential hematopoetic effects.

We have previously reported effects of maternal cadmium exposure on 5-methylcytosine methylation in the same cohort of newborns.^7^ However, the cadmium-associated sites were not overlapping with those identified here and adjustment for cadmium did not influence the result. Betel chewing was not influential in this study on arsenic, which might be owing to the fact that very few of the mothers mixed betel leaves with tobacco. Further adjustments for SES or other potential influential factors did not alter the associations; however, we cannot completely exclude that there might be residual confounding. A potential caveat of this study is that we were not able to sort cells in the blood samples during the field studies. We therefore measured DNA methylation in cord blood mononuclear cells, which are a mixture of different cell types with partly different methylation patterns. Thus, we cannot exclude that a potential cell-specific effect of arsenic might blur associations between DNA methylation and arsenic exposure. However, in the recent study by Koestler *et al*.,^30^ white blood cell distributions explained only a small proportion of the variability in patterns of cord blood DNA methylation associated with maternal arsenic exposure (3% for total arsenic in urine). Furthermore, some results from the Ingenuity Pathway Analysis (IPA) analysis, although plausible, should be carefully interpreted as a recent paper describes that certain diseases and pathways, for example, cancer and developmental-related pathways, tend to be overrepresented in the IPA.^53^

In conclusion, we show a clear relationship between maternal exposure to arsenic, especially in early pregnancy, and epigenetic effects early in life, particularly in boys. How that relates to the observed health effects of arsenic in early and later life remains to be elucidated. Our results emphasize the need to evaluate epigenetic effects of fetal environmental factors by sex and critical windows of exposure.

## Supplementary Material

Supplementary material To view supplementary material for this article, please visit http://dx.doi.org/10.1017/S2040174414000221.
