# Supplementary material for: Arsenic exposure in early pregnancy alters genome-wide DNA methylation in cord blood, particularly in boys
Source: J Dev Orig Health Dis. 2014 Apr 14;5(4):288–98. doi: 10.1017/S2040174414000221 (PMC4283288; doi:10.1017/S2040174414000221)
Supplement: Supplementary material — To view supplementary material for this article, please visit http://dx.doi.org/10.1017/S2040174414000221. [file S2040174414000221sup001.docx]

Supplemental Material for:

**Arsenic exposure in early pregnancy alters genome-wide DNA methylation in cord blood particularly in boys**

Karin Broberg, Sultan Ahmed, Karin Engström, Mohammad Bakhtiar Hossain, Simona Jurkovic Mlakar, Matteo Bottai, Margaretha Grandér, Rubhana Raqib, Marie Vahter

**Table of contents:**

Supplementary Figure S1. Principal component analysis of cord blood DNA methylation and characteristics of the mothers and their newborns.

Supplementary Figure S2. Distribution of the p-values for the coefficient associated with maternal urinary arsenic concentrations in early gestation from analysis of CpG methylation (n=482,421 sites).

Supplementary Table S1. The top 20 CpG sites with the strongest associations with urinary arsenic in gestational week 8.

Supplementary Table S2. SNPs in the probes for the top correlated CpG sites.

Supplementary Table S3. Analysis of the association between mother’s arsenic exposure and DNA methylation stratified by % of MMA.

Supplementary Table S4. Cancer-related genes associated with arsenic exposure in boys.

**Supplementary** **Figure S1. Principal component analysis of cord blood DNA methylation and characteristics of the mothers and their newborns. ^a^**Food group: the women received supplementation of both food and different micronutrients resulting in six different groups. Body mass index (BMI) is shown at gestational week (GW) 8, SES means socioeconomic status, arsenic exposure is total concentrations of arsenic metabolites in maternal urine, DNA concentration is the input amount on the plate and betel nut chewing is divided into three groups (no, yes - without tobacco added, and yes - with tobacco added).

**Supplementary** **Figure S2. Distribution of the p-values for the coefficient associated with maternal urinary arsenic concentrations in early gestation from linear regression analysis of CpG methylation (n=482,421 sites) in cord blood from all children.**

**Supplementary** **Table S1. Top 20 CpG sites in all newborns with the strongest associations (Spearman's rank correlation coefficient, R_s_) with maternal urinary arsenic concentrations in gestational week (GW) 8.^a^**

| **CpG sites** | **Chr** | **Gene** | **R_S_,**  **GW8** | **p**  **GW8** | **p FDR**  **GW8** | **R_S_, GW30** | **p**  **GW30** | **p FDR**  **GW30** |
| --- | --- | --- | --- | --- | --- | --- | --- | --- |
| cg23385248 | 15 |  | -0.47 | 3*10^-08^ | 0.01 | -0.29 | 0.001 | 1 |
| cg16810054 | 20 | *TP53INP2* | -0.44 | 3*10^-07^ | 0.06 | -0.10 | 0.3 | 1 |
| cg02895582 | 8 |  | -0.43 | 5*10^-07^ | 0.06 | -0.23 | 0.01 | 1 |
| cg01758314 | 17 | *ALOX12P2* | 0.43 | 6*10^-07^ | 0.06 | 0.15 | 0.09 | 1 |
| cg10804656 | 10 |  | -0.43 | 6*10^-07^ | 0.06 | -0.15 | 0.09 | 1 |
| cg06588605 | 20 | *TGIF2* | -0.43 | 8*10^-07^ | 0.06 | -0.16 | 0.08 | 1 |
| cg11597969 | 13 |  | -0.42 | 1*10^-06^ | 0.06 | -0.17 | 0.06 | 1 |
| cg11712459 | 18 |  | -0.42 | 1*10^-06^ | 0.06 | -0.23 | 0.009 | 1 |
| cg08057038 | 11 | *MUPCDH* | -0.42 | 1*10^-06^ | 0.06 | -0.20 | 0.02 | 1 |
| cg07116631 | 16 |  | -0.42 | 1*10^-06^ | 0.06 | -0.19 | 0.03 | 1 |
| cg13983776 | 19 | *ZNF761* | -0.41 | 1*10^-06^ | 0.06 | -0.24 | 0.005 | 1 |
| cg01763990 | 2 | *RGPD4* | -0.41 | 2*10^-06^ | 0.06 | -0.23 | 0.008 | 1 |
| cg08054244 | 14 | *KCNK10* | -0.41 | 2*10^-06^ | 0.06 | -0.25 | 0.004 | 1 |
| cg16267304 | 21 | *HSF2BP* | -0.41 | 2*10^-06^ | 0.06 | -0.23 | 0.007 | 1 |
| cg19497517 | 8 | *PLEC1* | -0.41 | 2*10^-06^ | 0.06 | -0.23 | 0.009 | 1 |
| cg09606015 | 3 | *ATP11B* | -0.41 | 2*10^-06^ | 0.06 | -0.16 | 0.07 | 1 |
| cg13659051 | 9 | *LRRC26* | -0.41 | 2*10^-06^ | 0.06 | -0.15 | 0.07 | 1 |
| cg26908876 | 11 | *KCNQ1OT1* | -0.40 | 3*10^-06^ | 0.07 | -0.21 | 0.01 | 1 |
| cg01762091 | 17 | *YBX2* | -0.40 | 3*10^-06^ | 0.07 | -0.34 | 8*10^-05^ | 1 |
| cg19304273 | 11 |  | -0.40 | 3*10^-06^ | 0.07 | -0.31 | <0.001 | 1 |

^a^For comparison the same sites are shown in gestational week (GW) 30. Abbreviations: Chr., Chromosome number, False Discovery Rate (FDR). p-value adjusted for False Discovery Rate (FDR).

**Supplementary** **Table S2. SNPs in the probes for the top 20 correlated CpG sites reported in Table 2 (main text).**

| **CpG sites** | **Chr.** | **Gene** | **Probe_SNP^a^** | **Probe_SNP_10**^b^ | **MAF** | **MAF source** |
| --- | --- | --- | --- | --- | --- | --- |
| ***Boys*** | | | | | | |
| cg15255455 | 19 | *PLIN5* |  |  |  |  |
| cg13659051 | 9 | *LRRC26* |  |  |  |  |
| cg17646418 | 6 | *RPS6KA2* | rs7758528 (G/A) |  | G=1 | JPT^c^ |
| cg02975107 | 17 |  |  |  |  |  |
| cg00592460 | 6 |  |  |  |  |  |
| cg05114100 | X | *LUZP4* |  |  |  |  |
| cg23727087 | 12 |  |  |  |  |  |
| cg17158414 | 2 | *KRTCAP3* |  |  |  |  |
| cg24042517 | 17 | *HOXB9* |  |  |  |  |
| cg12124733 | X | *DDX26B* |  |  |  |  |
| cg19802390 | 1 |  |  |  |  |  |
| cg24456094 | 6 | *MDGA1* |  |  |  |  |
| cg15479387 | 6 | *HLA-F-AS1* |  |  |  |  |
| cg25938735 | 8 |  |  |  |  |  |
| cg20415517 | 11 | *BRSK2* |  |  |  |  |
| cg12094808 | 15 | *FRMD5* | rs76847582 (C/G) |  | G=0.046^d^ | 1000 Genomes^d^ |
| cg14920289 | 14 | *SLC22A17* |  |  |  |  |
| cg21097283 | 5 | *OTP* |  |  |  |  |
| cg17322444 | 19 | *GPATCH1* |  |  |  |  |
| cg24680320 | 19 |  | rs59325648 (G/C) |  | G=0.017 | 1000 Genomes |
| ***Girls*** | | | | | | |
| cg22614624 | 2 | *DTYMK* |  | rs7369612 (G/A) | A=0.349 | 1000 Genomes |
| cg08421080 | 12 | *RIMBP2* | rs11834732 (T/C) |  | NA |  |
| cg02240066 | 2 |  |  |  |  |  |
| cg09606015 | 3 | *ATP11B* |  |  |  |  |
| cg06411879 | 10 | *NEBL* |  |  |  |  |
| cg23385248 | 15 |  |  |  |  |  |
| cg08962682 | 12 | *RIMKLB* |  |  |  |  |
| cg05173913 | 17 | *C17orf50* |  |  |  |  |
| cg21587006 | 10 |  |  |  |  |  |
| cg06584028 | 2 | *GFPT1* | rs71712861 |  | NA |  |
| cg04361852 | 11 | *UCP2* |  |  |  |  |
| cg00384577 | 19 | *ITPKC* |  |  |  |  |
| cg14862981 | 5 |  |  |  |  |  |
| cg09005548 | 6 | *ZBTB22* |  |  |  |  |
| cg14673387 | 10 |  |  |  |  |  |
| cg22328208 | 8 | *TSPYL5* |  |  |  |  |
| cg07276007 | 11 | *AP2A2* |  |  |  |  |
| cg07936037 | 6 | *SSR1* |  |  |  |  |
| cg26726230 | 17 | *SLC38A10* |  |  |  |  |
| cg01094684 | 19 | *UQCR* |  |  |  |  |

^a^Probe_SNP: Assays with SNPs within probe >10bp from query site

^b^Probe_SNP_10: Assays with SNPs within probe ≤10bp from query site

^c^In Japanese in Tokyo, Japan (JPT) http://ccr.coriell.org/Sections/Collections/NHGRI/1000Japanese.aspx?PgId=703&coll=HG

^d^MAF (Minor Allele Frequency) reported in 1000 Genomes database: http://www.1000genomes.org/node/506

**Supplementary** **Table S3. Linear regression analysis of association between arsenic concentrations and cord blood DNA methylation for the top20 CpG sites in boys and girls, stratified by the fraction of the monomethylated arsenic metabolite (%MMA) in urine.^a^**

| **CpG sites** | **Chr** | **Gene** | **Total arsenic in maternal urine at gestational week 8 (log2-transformed)** | | | |
| --- | --- | --- | --- | --- | --- | --- |
|  |  |  | **Low % of MMA** | | **High % of MMA** | |
|  |  |  | **B** | **P value** | **B (95%CI)** | **P** |
| ***Boys*** | | | | | | |
| cg15255455 | 19 | *PLIN5* | -0.011 (-0.022, 0.00005) | 0.05 | -0.020 (-0.030, -0.0097) | 0.001 |
| cg13659051 | 9 | *LRRC26* | -0.013 (-0.023, -0.0021) | 0.02 | -0.022 (-0.035, -0.0075) | 0.005 |
| cg17646418 | 6 | *RPS6KA2* | -0.0090 (-0.015, -0.0030) | 0.005 | -0.015 (-0.027, -0.0030) | 0.02 |
| cg02975107 | 17 |  | -0.0093 (-0.017, -0.0016) | 0.02 | -0.014 (-0.021, -0.0062) | 0.002 |
| cg00592460 | 6 |  | -0.021 (-0.030¸ -0.010) | <0.001 | -0.022 (-0.039, -0.0053) | 0.01 |
| cg05114100 | X | *LUZP4* | -0.015 (-0.024, -0.0051) | 0.004 | -0.013 (-0.023, -0.0020) | 0.02 |
| cg23727087 | 12 |  | -0.0067 (-0.012, -0.0009) | 0.02 | -0.011 (-0.019, -0.0026) | 0.01 |
| cg17158414 | 2 | *KRTCAP3* | -0.024 (-0.038, -0.0092) | 0.002 | -0.021 (-0.049, 0.0083) | 0.15 |
| cg24042517 | 17 | *HOXB9* | -0.0075 (-0.013, -0.0012) | 0.02 | -0.017 (-0.024, -0.0086) | <0.001 |
| cg12124733 | X | *DDX26B* | 0.012 (0.00085, 0.022) | 0.04 | 0.0029 (-0.00044, 0.0063) | 0.08 |
| cg19802390 | 1 |  | -0.023 (-0.040, -0.0053) | 0.01 | -0.020 (-0.040, -0.00034) | 0.05 |
| cg24456094 | 6 | *MDGA1* | -0.0097 (-0.01, -0.000012) | 0.05 | -0.011 (-0.019, -0.0018) | 0.02 |
| cg15479387 | 6 | *LOC285830* | -0.0042 (-0.007, -0.00098) | 0.01 | -0.0052 (-0.0087, -0.0016) | 0.007 |
| cg25938735 | 8 |  | -0.014 (-0.022, -0.0055) | 0.002 | -0.018 (-0.028, -0.0074) | 0.002 |
| cg20415517 | 11 | *BRSK2* | -0.0086 (-0.014, -0.0031) | 0.003 | -0.018 (-0.032, -0.0040) | 0.02 |
| cg12094808 | 15 | *FRMD5* | -0.0095 (-0.017, -0.0011) | 0.03 | -0.012 (-0.020, -0.0028) | 0.01 |
| cg14920289 | 14 | *SLC22A17* | -0.0076 (-0.014, -0.0010) | 0.02 | -0.016 (-0.022, -0.0096) | <0.001 |
| cg21097283 | 5 | *OTP* | 0.0069 (0.00056, 0.013) | 0.03 | 0.010 (0.00035, 0.020) | 0.04 |
| cg17322444 | 19 | *GPATCH1* | 0.0043 (0.0019, 0.0066) | 0.001 | 0.0032 (-0.00020, 0.0067) | 0.06 |
| cg24680320 | 19 |  | -0.0032 (-0.0055, -0.0010) | 0.006 | -0.0032 (-0.0044, -0.0019) | <0.001 |
| ***Girls*** | | | | | | |
| cg22614624 | 2 | *DTYMK* | 0.0034 (-0.013, 0.020) | 0.7 | 0.014 (0.0053, 0.022) | 0.002 |
| cg08421080 | 12 | *RIMBP2* | -0.0034 (-0.021, 0.014) | 0.7 | 0.011 (0.0018, 0.019) | 0.02 |
| cg02240066 | 2 |  | -0.0053 (-0.014, 0.0034) | 0.2 | -0.0051 (-0.0076, -0.0024) | <0.001 |
| cg09606015 | 3 | *ATP11B* | -0.013 (-0.021, -0.0039) | 0.007 | -0.0058 (-0.010, -0.00096) | 0.02 |
| cg06411879 | 10 | *NEBL* | 0.014 (-0.0076, 0.034) | 0.2 | 0.011 (0.0014, 0.020) | 0.03 |
| cg23385248 | 15 |  | -0.011 (-0.028, 0.0071) | 0.2 | -0.014 (-0.022, -0.0053) | 0.002 |
| cg08962682 | 12 | *RIMKLB* | -0.0028 (-0.0088, 0.0031) | 0.3 | -0.0031 (-0.0059, -0.00018) | 0.04 |
| cg05173913 | 17 | *C17orf50* | -0.00098 (-0.0077, 0.0058) | 0.8 | -0.0067 (-0.013, -0.00041) | 0.04 |
| cg21587006 | 10 |  | 0.15 (0.035, 0.26) | 0.01 | 0.028 (-0.037, 0.093) | 0.4 |
| cg06584028 | 2 | *GFPT1* | -0.0078 (-0.015, -0.00049) | 0.04 | -0.0049 (-0.0079, -0.0020) | 0.002 |
| cg04361852 | 11 | *UCP2* | -0.0065 (-0.014, 0.0011) | 0.09 | -0.0032 (-0.0064, -0.000024) | 0.05 |
| cg00384577 | 19 | *ITPKC* | -0.0027 (-0.0089, 0.0036) | 0.4 | -0.0053 (-0.0084, -0.0021) | 0.002 |
| cg14862981 | 5 |  | -0.0051 (-0.011, 0.0011) | 0.1 | -0.0020 (-0.0041, 0.00016) | 0.07 |
| cg09005548 | 6 | *ZBTB22* | 0.0085 (-0.0085, 0.025) | 0.3 | 0.011 (0.0056, 0.017) | <0.001 |
| cg14673387 | 10 |  | -0.0038 (-0.0080, 0.00054) | 0.08 | -0.0029 (-0.0047, -0.0010) | 0.003 |
| cg22328208 | 8 | *TSPYL5* | -0.026 (-0.055, 0.0032) | 0.08 | -0.012 (-0.024, -0.00020) | 0.05 |
| cg07276007 | 11 | *AP2A2* | 0.0019 (-0.0059, 0.0097) | 0.6 | 0.0069 (0.0015, 0.012) | 0.01 |
| cg07936037 | 6 | *SSR1* | -0.000015 (-0.0015, 0015) | 1.0 | -0.0018 (-0.0031, -0.00042) | 0.01 |
| cg26726230 | 17 | *SLC38A10* | 0.0054 (-0.0088¸ 0.019) | 0.5 | 0.014 (0.0025, 0.025) | 0.02 |
| cg01094684 | 19 | *UQCR* | -0.00077 (-0.0026, 0.0011) | 0.4 | -0.0011 ( -0.0026, 0.00053) | 0.2 |

^a^MMA– methylarsonic acid in maternal urine; median split at 9.3% of total arsenic metabolites in urine. Regression analyses were adjusted for mother age, BMI, gestational age at birth, SES, exact gestational weeks at urine collection. Exposure variable is log2-transformed

**Supplementary** **Table S4. Cancer-related genes (top 50) associated with arsenic exposure in boys.^a^**

| **Gene** | **Entrez Gene Name** | **Rs** |
| --- | --- | --- |
| *LRRC26* | leucine rich repeat containing 26 | -0.62 |
| *HOXB9* | homeobox B9 | -0.57 |
| *BRSK2* | BR serine/threonine kinase 2 | -0.56 |
| *ASB2* | ankyrin repeat and SOCS box containing 2 | -0.55 |
| *FRK* | fyn-related kinase | -0.53 |
| *TNXB* | tenascin XB | -0.53 |
| *DPPA2* | developmental pluripotency associated 2 | -0.53 |
| *GCDH* | glutaryl-CoA dehydrogenase | -0.53 |
| *MACROD2* | MACRO domain containing 2 | -0.53 |
| *FGF18* | fibroblast growth factor 18 | -0.53 |
| *CSMD1* | CUB and Sushi multiple domains 1 | -0.52 |
| *CIRBP* | cold inducible RNA binding protein | -0.52 |
| *STK3* | serine/threonine kinase 3 | -0.52 |
| *DDX39A* | DEAD (Asp-Glu-Ala-Asp) box polypeptide 39A | -0.52 |
| *PCDHA2* | protocadherin alpha 2 | -0.52 |
| *TGIF2* | TGFB-induced factor homeobox 2 | -0.52 |
| *RGPD5* | RANBP2-like and GRIP domain containing 5 | -0.52 |
| *RNF213* | ring finger protein 213 | -0.52 |
| *HLX* | H2.0-like homeobox | -0.51 |
| *ADARB2* | adenosine deaminase, RNA-specific, B2 | -0.51 |
| *ABCC4* | ATP-binding cassette, sub-family C (CFTR/MRP), member 4 | -0.51 |
| *DIP2C* | DIP2 disco-interacting protein 2 homolog C (Drosophila) | -0.51 |
| *FZD5* | frizzled family receptor 5 | -0.51 |
| *GRIN2A* | glutamate receptor, ionotropic, N-methyl D-aspartate 2A | -0.51 |
| *GNAQ* | guanine nucleotide binding protein (G protein), q polypeptide | -0.51 |
| *MCF2L* | MCF.2 cell line derived transforming sequence-like | -0.51 |
| *TUBB4A* | tubulin, beta 4A class IVa | -0.51 |
| *PRAME* | preferentially expressed antigen in melanoma | -0.50 |
| *TTLL1* | tubulin tyrosine ligase-like family, member 1 | -0.50 |
| *VAV2* | vav 2 guanine nucleotide exchange factor | -0.50 |
| *SLC2A14* | solute carrier family 2 (facilitated glucose transporter), member 14 | -0.50 |
| *DIO1* | deiodinase, iodothyronine, type I | -0.50 |
| *GRIN2D* | glutamate receptor, ionotropic, N-methyl D-aspartate 2D | -0.50 |
| *ASPSCR1* | alveolar soft part sarcoma chromosome region, candidate 1 | -0.50 |
| *NFE2L2* | nuclear factor (erythroid-derived 2)-like 2 | -0.50 |
| *SFRP2* | secreted frizzled-related protein 2 | -0.50 |
| *PTPRN2* | protein tyrosine phosphatase, receptor type, N polypeptide 2 | -0.50 |
| *LIMA1* | LIM domain and actin binding 1 | -0.50 |
| *AATK* | apoptosis-associated tyrosine kinase | -0.49 |
| *BCL11B* | B-cell CLL/lymphoma 11B (zinc finger protein) | -0.49 |
| *CBX7* | chromobox homolog 7 | -0.49 |
| **Supplementary** **Table S4. Cancer-related genes associated with arsenic exposure in boys. Continued** | | |
| *PSMB2* | proteasome (prosome, macropain) subunit, beta type, 2 | -0.49 |
| *CSF3* | colony stimulating factor 3 (granulocyte) | -0.49 |
| *WWOX* | WW domain containing oxidoreductase | -0.49 |
| *PARD6A* | par-6 partitioning defective 6 homolog alpha (C. elegans) | -0.49 |
| *PDRG1* | p53 and DNA-damage regulated 1 | -0.49 |
| *CELF4* | CUGBP, Elav-like family member 4 | -0.49 |
| *GNAS* | GNAS complex locus | -0.49 |
| *KLK10* | kallikrein-related peptidase 10 | -0.48 |
| *MEST* | mesoderm specific transcript homolog (mouse) | -0.48 |

^a^Spearman correlation’s (Rs) between beta values of cord blood DNA methylation and maternal arsenic concentrations in urine.
